# Structural characterization of *copia-*type retrotransposons leads to insights into the marker development in a biofuel crop, *Jatropha curcas* L.

**DOI:** 10.1186/1754-6834-6-129

**Published:** 2013-09-10

**Authors:** Atefeh Alipour, Suguru Tsuchimoto, Hiroe Sakai, Nobuko Ohmido, Kiichi Fukui

**Affiliations:** 1 Department of Biotechnology, Graduate School of Engineering, Osaka University, Suita, Osaka 565-0871, Japan; 2 Plant Bioengineering for Bioenergy Laboratory, Graduate School of Engineering, Osaka University, Suita, Osaka 565-0871, Japan; 3 Graduate School of Human Development and Environment, Kobe University, Kobe, Hyogo 657-8501, Japan

**Keywords:** *Jatropha curcas* L, *copia*-type retrotransposon, Retrotransposon-based insertion polymorphism (RBIP), Fluorescence *in situ* hybridization (FISH)

## Abstract

**Background:**

Recently, *Jatropha curcas* L. has attracted worldwide attention for its potential as a source of biodiesel. However, most DNA markers have demonstrated high levels of genetic similarity among and within jatropha populations around the globe. Despite promising features of *copia*-type retrotransposons as ideal genetic tools for gene tagging, mutagenesis, and marker-assisted selection, they have not been characterized in the jatropha genome yet. Here, we examined the diversity, evolution, and genome-wide organization of *copia*-type retrotransposons in the Asian, African, and Mesoamerican accessions of jatropha, then introduced a retrotransposon-based marker for this biofuel crop.

**Results:**

In total, 157 PCR fragments that were amplified using the degenerate primers for the reverse transcriptase (RT) domain of *copia*-type retroelements were sequenced and aligned to construct the neighbor-joining tree. Phylogenetic analysis demonstrated that isolated *copia* RT sequences were classified into ten families, which were then grouped into three lineages. An in-depth study of the jatropha genome for the RT sequences of each family led to the characterization of full consensus sequences of the jatropha *copia*-type families. Estimated copy numbers of target sequences were largely different among families, as was presence of genes within 5 kb flanking regions for each family. Five *copia*-type families were as appealing candidates for the development of DNA marker systems. A candidate marker from family *Jc7* was particularly capable of detecting genetic variation among different jatropha accessions. Fluorescence *in situ* hybridization (FISH) to metaphase chromosomes reveals that *copia*-type retrotransposons are scattered across chromosomes mainly located in the distal part regions.

**Conclusion:**

This is the first report on genome-wide analysis and the cytogenetic mapping of *copia*-type retrotransposons of jatropha, leading to the discovery of families bearing high potential as DNA markers. Distinct dynamics of individual *copia*-type families, feasibility of a retrotransposon-based insertion polymorphism marker system in examining genetic variability, and approaches for the development of breeding strategies in jatropha using *copia*-type retrotransposons are discussed.

## Background

The increased global demand for energy and unstable petroleum prices, coupled with environmental awareness to reduce CO_2_ emission, has become a comprehensive movement toward the transitioning from fossil to renewable fuels such as biofuels. Although biofuels offer a diverse range of promising alternatives, a spurt in world population growth and concerns over food security have reawakened interest in the development of non-edible vegetative oleaginous resources such as jatropha [1,2]. The jatropha (*Jatropha curcas* L.) is a shrub belongs to the *Euphorbiaceae* family. It is native to Mexico and Central America [3], and is now propagated in tropical and subtropical areas of Asia, Africa, and Latin America [4]. The oil content of jatropha seeds, ranging from 40-60% oil by dry weight, has the highest level among oil-bearing tree species, which gives this plant the highest potential as a raw material for the biodiesel production [5]. Jatropha is a vigorous drought-resistant crop that can grow on barren lands with a low level of greenhouse gas emission, and therefore its cultivation does not compete with food crops production [6,7]. Moreover, processing jatropha oil results in some byproducts that can be used as raw materials to produce plastic, organic fertilizer, synthetic fiber, and animal feed [8,9].

Jatropha is a diploid plant with 22 chromosomes and a genome size of approximately 370 Mb [10]. The current lack of comprehensive genetic information about variation of jatropha makes it difficult to produce commercial lines. Phenotype-based selection from local germplasms of Asia and Africa, neither of which is the origin of the species, may lead to high inbreeding in jatropha populations with low genetic diversity. Therefore, the global evaluation of genetic structure in existing jatropha populations, including those of Mexico or Central America, is necessary for marker-assisted selection to breed and introduce the commercial lines. Although the whole genome sequence of jatropha has been opened in our previous studies [11,12], relatively little is known about the genetic variability and population dynamics of this oil crop. Most of the earlier studies revealed a high genetic similarity among populations using DNA markers such as RAPD, AFLP, SSR, and ISSR [13-17]. Therefore, it seems necessary to identify more powerful markers to assess genetic variations in this energy crop.

Given their activity in driving genome diversification, retrotransposons have been recently exploited as more informative molecular markers to assess genetic diversity and the marker-assisted selection of plant species in various ways [18]. The retrotransposon is one of two major groups of eukaryotic transposable elements that copy themselves visa RNA intermediates, leading to various gene regulation, speciation, and variation among identical population [19,20]. Variance in copy number over a relatively short evolutionary timescale serves retrotransposons as a key component of the structural evolution in plant genomes [21]. Retrotransposon-based markers are ubiquitous, co-dominant and, more importantly, irreversible. The utility of transposon-based marker systems has been widely proven in phylogenetic, genetic diversity, breeding, and mapping studies in various crop plants and tree species, due to their easy detection by a simple PCR [18]. Of these types of markers, retrotransposon-based insertion polymorphism (RBIP) has been the most affordable and developed for high-throughput applications [22]. These characteristics make them as perfect molecular markers for genetic studies including DNA fingerprinting, phylogenetic studies, and marker-assisted selection for plant breeding [18,22,23].

Based on the presence of long terminal repeats (LTRs) that surround the internal region, retrotransposons are classified into two types: LTR and non-LTR retrotransposons. LTR retrotransposons accumulate in plant genomes ranging between 40-70% of the total genomic DNA [24]. They have two regions encoding the group-specific antigen (Gag) domain and the polyprotein of retroviruses (Pol) domain, respectively. The *pol* region is comprised of four genes, which encode four enzymes—protease, integrase, reverse transcriptase, and RNase H—that are essential to disassembling the Pol polyprotein and driving the retrotransposition [25]. LTR retrotransposons consist of two major types, *copia* and *gypsy*[26], both of which are further subdivided into numerous families. Families of plant *copia*-type retrotransposons were classified into six lineages [27].

Fluorescence *in situ* hybridization (FISH) studies of plant chromosomes showed that LTR-retrotransposon elements were frequently widespread across the chromosomes. The *copia*-type tends to accumulate in the distal part of chromosomes, whereas *gypsy*-type elements prefer the centromeric region [28]. In some cases, however, the converse distribution pattern has been reported [28,29]. Therefore, according to higher gene density in the distal parts of chromosomes than in their centromeric regions, the localization of retrotransposons is a key factor for molecular marker selection. The aim of this study is to characterize the heterogeneity and chromosomal distribution of *copia-*type retrotransposons in the jatropha genome to gain new insights into the population genetics with the goal of finally finding informative markers for breeding new, genetically improved jatropha varieties.

## Results

### Identification of *copia*-type RT sequences in the jatropha genome

In order to detect *copia*-type retrotransposons in the genome of jatropha (*Jatropha curcas* L.), a degenerate primer set corresponding to conserved sequences of the *copia*-type RT gene of higher plants [30] was exploited to amplify DNA fragments of approximately 300 bp. The amplified PCR products were cloned, and at least 10 randomly selected clones from each of the five lines of jatropha (from the Philippines, China, Thailand, Indonesia, and Uganda) were sequenced. Two other degenerate primer sets [31,32] were also used to amplify the RT sequence from Philippine and Chinese lines. With all primer sets, various *copia*-type retrotransposons were verified, and about 98% of the fragments had sequences identifiable as the *copia*-type RT region. In total, 157 resulting sequences were used for the phylogenetic analysis. All of the sequences, except two, were different from each other.

### Classification of identified RT sequences

**Figure 1:**
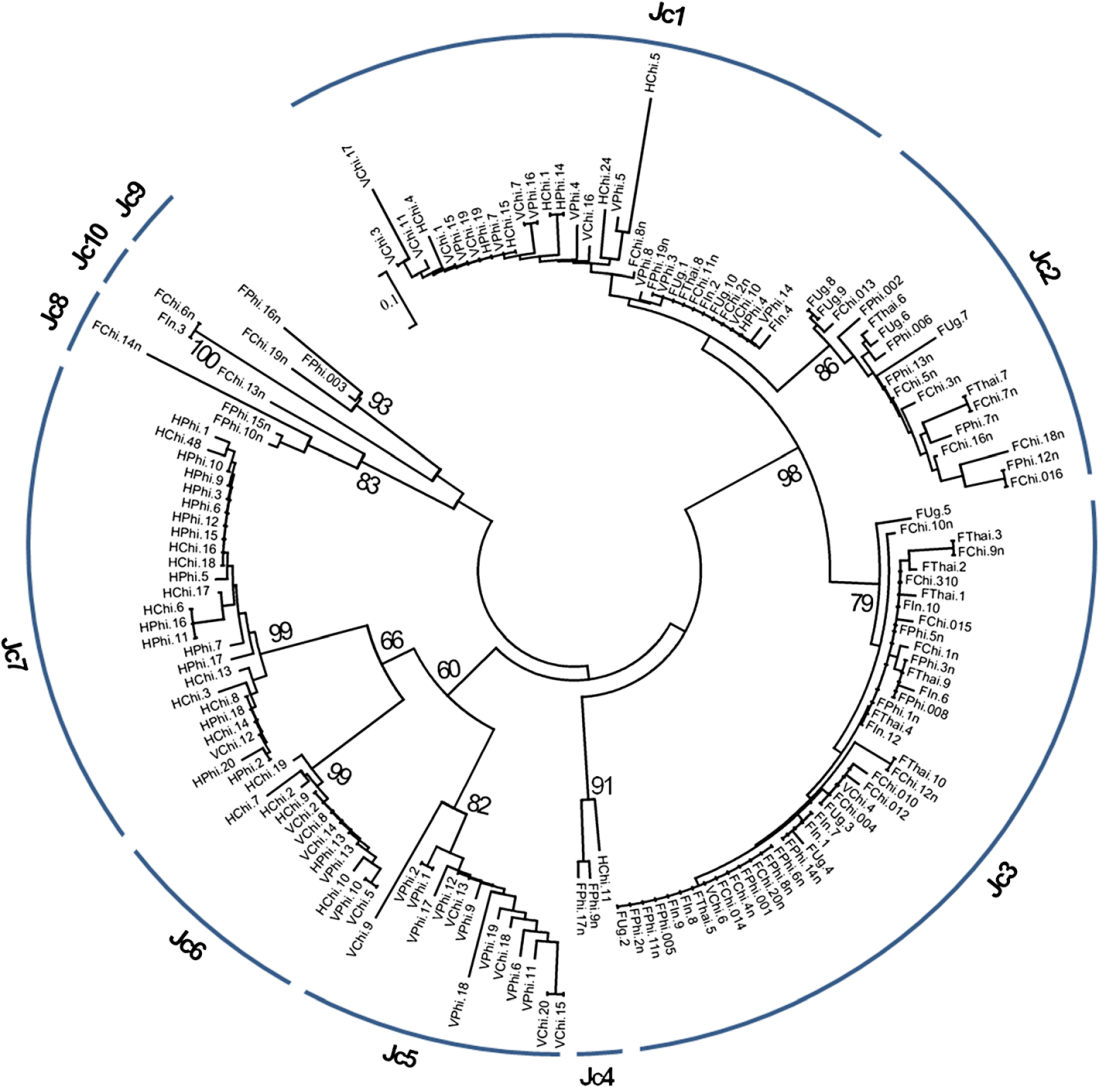
**Neighbor-joining dendrogram of conserved region of *copia*-type reverse transcriptase (RT) gene isolated from five accessions of jatropha.** (F: RT isolated by F primer set, V: RT isolated by V primer set, H: RT isolated by H primer set, Phi: Philippine accession, Chi: Chinese accession, Thai: Thai accession, Ug: Ugandan accession In: Indonesian).

Amino-acid sequences encoded by all identified RT nucleotide sequences were deduced with a consideration of spontaneous frameshift mutations and trimmed to their overlapped region of about 75 amino acids in length. To observe divergence, the RT sequences were aligned, and a phylogenetic tree was constructed by the Neighbor-Joining method. High sequence heterogeneity was observed among the RT sequences, and they could be classified into ten distinct groups (Figure 1). It was thus inferred the presence of at least ten distinct *copia*-type families in the jatropha genome. There were then nominated as Jc (Jatropha *copia*-type retrotransposon) and numbered them from 1 to 10. The isolated RT clone numbers in each family were counted and the family distribution patterns were compared among five jatropha lines (Additional file 1). We found that distribution patterns were not significantly different from each other among the five jatropha lines. This suggests that no evident bursts of amplification occurred in either of the identified *copia*-type families after the divergence of the five lines. Likewise, the different distribution patterns in one primer set compared to that of made by other primer sets might be owing to the respective amplification biases in PCR.

**Figure 2:**
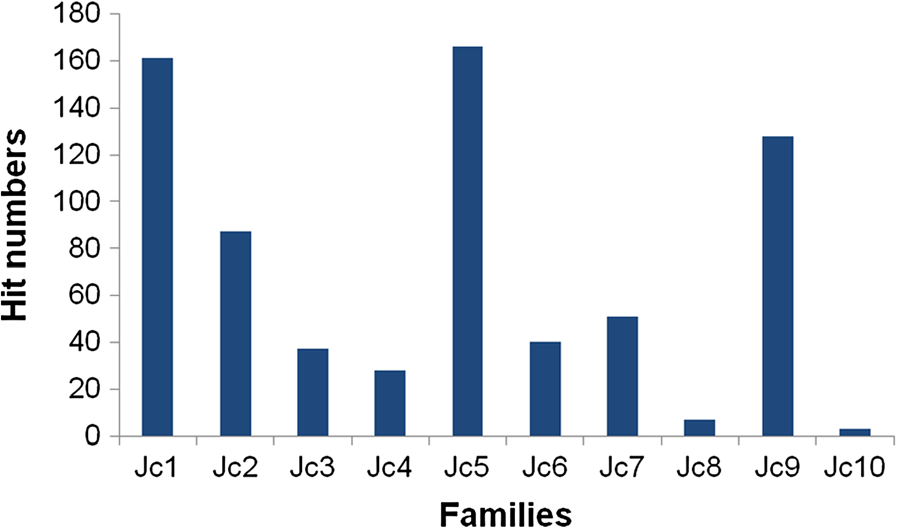
**Relative frequency of isolated *copia*-type RT families in the genome of *J. curcas*.** Hit numbers of BLAST search (E<e^-20^) of the Jatropha Genome Database using the consensus RT sequences as queries were shown.

The consensus of RT sequences of each family was inferred by using the jatropha genome database (http://www.kazusa.or.jp/jatropha/). To estimate relative copy numbers of the ten *copia*-type families of jatropha, a BLAST searching of the jatropha genome database was performed using the consensus RT sequences. Three families, *Jc1*, 5, and 9, showed the highest hit numbers: 161, 166, and 128, respectively (Figure 2). Our data indicated that these three families have higher copy numbers in the jatropha genome than other families. In contrast, *Jc8* and *Jc10* showed low hit numbers, 7 and 3, implying their low copy numbers in the genome of jatropha. The hit number of *Jc3* was relatively small, whereas this family had the highest number of isolated RT sequences by PCR (Figure 1). This observation might be owing to the presence of amplification bias in PCR using degenerate primers, as described above.

### Characterization of the *copia-*type families of jatropha

**Figure 3:**
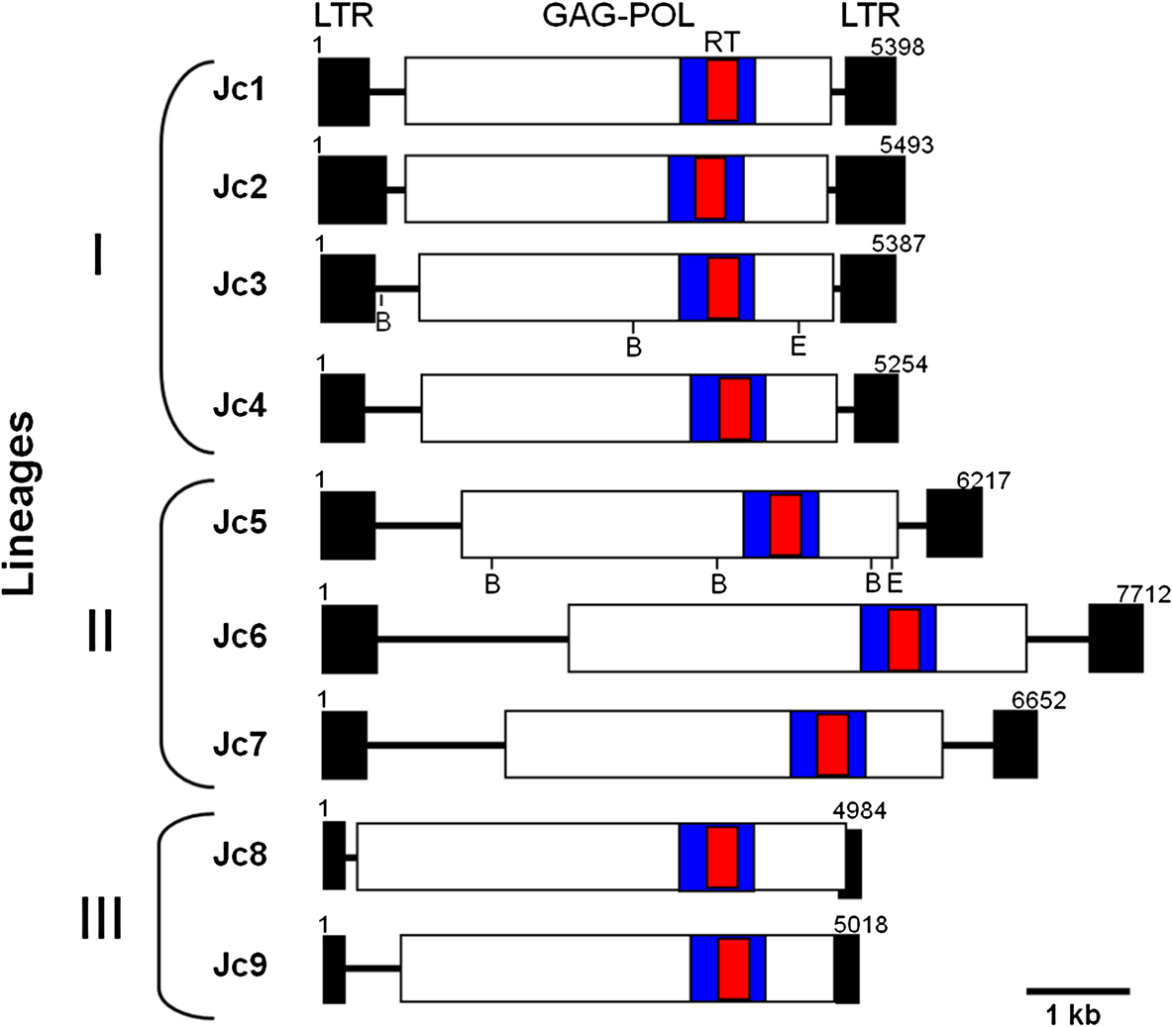
**Schematic structures of jatropha *copia*-type retrotransposons.** Structures of consensus sequences are shown with their LTRs (filled boxes) and the GAG-POL regions (open boxes). Regions of cloned RT sequences are shown by red boxes, and those used for the phylogenetic tree construction (see Figure 4) are shown by blue boxes. Lineages (I, II, and III) of the families are also indicated. *Eco*RI and *Bam*HI restriction sites of *Jc3* and *Jc5* sequences are shown below the structures. In family *Jc8* the POL region has exceeded into the right LTR. Numbers are correspondence of the consensus sequences.

A complete characterization of retrotransposons is required for accurate verification of their identity and phylogeny [18], as well as for marker development. In order to retrieve the full-length retrotransposon sequence of each family, the jatropha genome database was searched using consensus RT sequences as queries, and contigs with significant sequence similarities were used for multiple sequence alignment. Aligning the contigs corresponding to each family enabled us to deduce their relevant consensus sequences including long terminal repeats, except for *Jc10*, which had a low copy number for deducing the consensus sequence. Schematic structures of the full consensus sequences of nine jatropha *copia*-type families are shown in Figure 3. The length of the full sequences varied from 7712 bp (*Jc6*) to 4984 bp (*Jc8*). All sequences had a PBS that was complementary to the tRNA^Met^ sequence (5′-TGGTATCAGAGC-3′), as well as PPT sequences (Table 1). Each of the nine sequences had an ORF encoding the GAG-POL polyprotein of 1299–1528 amino acids in length, which had conserved motifs of GAG, Protease, Integrase, and RNase H, as well as that of RT (data not shown).

**Table 1:** **Structural features of consensus sequences of *copia*-type retrotransposons in jatropha**

**Lineage**	**Family**	**Total length (bp)**	**LTR length (bp)**	**GAG-POL (aa)**	PBS*	PPT**
I	*Jc1*	5398	510	1339	agTGGTATCAGAGCC	AGGGGGAGAtt
I	*Jc2*	5493	697	1326	acTGGTATCAGAGCC	GGGAGGGGGAGAAt
I	*Jc3*	5387	523	1314	agTGGTATCAGAGCC	AGGGGGAGAtt
I	*Jc4*	5254	440	1299	acTGGTATCAGAGCC	AAGtGGGAGAt
II	*Jc5*	6217	474	1370	tgTGGTATCAGAGCC	AAGTGGGAGAt
II	*Jc6*	7712	959	1428	atTGGTATCAGAGCC	AAGtAGAGAAtGGA
II	*Jc7*	6652	447	1358	agTGGTATCAGAGCC	AAGtGGGAGAt
III	*Jc8*	4984	210	1528	TGGTATCAGAGCt	GAGGGGGAG
III	*Jc9*	5018	161	1398	TGGTATCAGAGCC	GAGGGGGAG

* Sequences complementary to the tRNA^Met^ sequence are shown in the upper case.

** Purine nucleotides were shown in the upper case.

**Figure 4:**
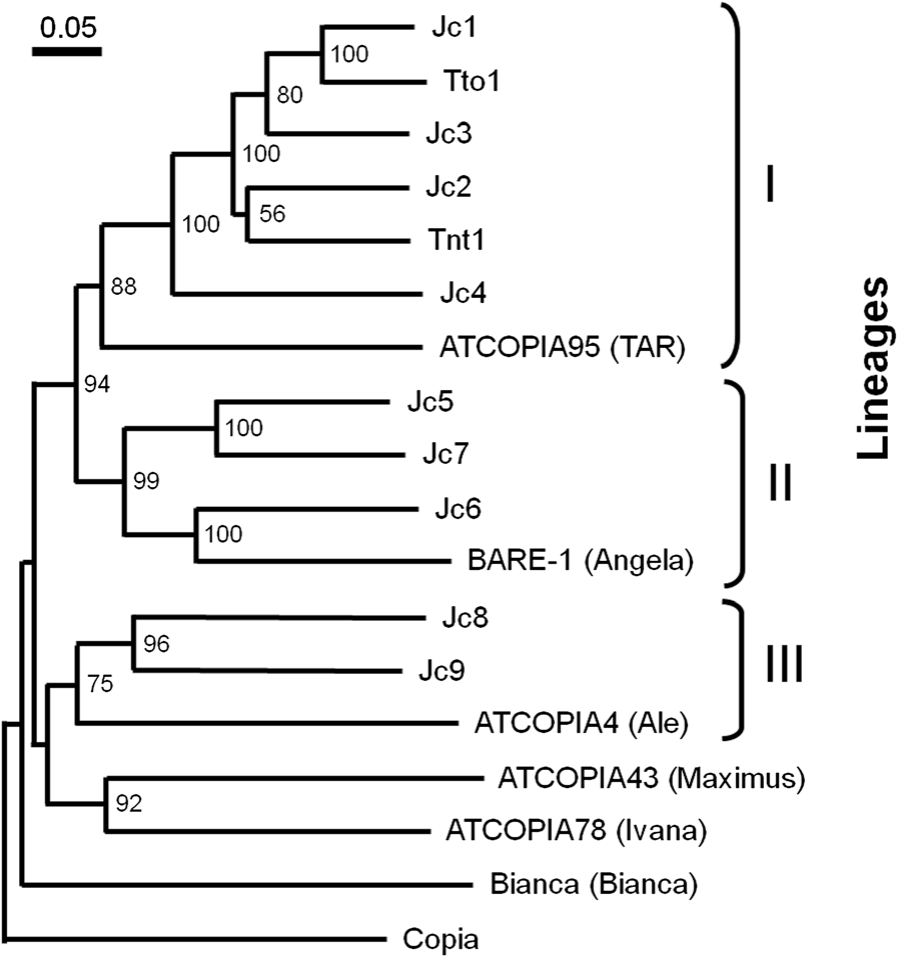
**Phylogenetic tree of consensus *copia *sequences.** The tree was constructed using RT sequences of *J. curcas* identified in this study and those of other plant species. An RT sequence of Drosophila *copia* was used as outgroup. Lineages of plant *copia*-type families reported by Wicker and Keller [27] were shown in parentheses. The bootstrap values over 50 were indicated at the nodes.

Conserved RT sequences (ca. 220 amino acids) of the GAG-POL polyprotein were aligned with those of representative *copia*-type elements in other plant species: *Tto1*, *Tnt1* of tobacco, *ATCOPIA4*, *43*, *78*, *95* of *Arabidopsis*, *BARE*-1 of barley, Bianca of wheat [27,33-35], and Drosopila *copia*[26]. A phylogenetic tree was constructed to assess the relationship between them (Figure 4). The tree indicates that there are three *copia*-type retrotransposon lineages in jatropha. Lineage I contains *Jc1*-*Jc4*, which are clustered with *Tto1* and *Tnt1*, active *copia*-type elements of tobacco, and *ATCOPIA95* of *Arabidopsis*. Notably, *Jc1* and *Tto1* are closely related to each other, and the sequence identity between them was 72% in the RT sequence applied to the tree (223 amino acids). Lineage II contains *Jc5*-*Jc7*, clustered with barley *BARE*-1, and Lineage III contains *Jc8*-*Jc9*, clustered with *ATCOPIA4* of *Arabidopsis*. The tree also suggests that Lineages I, II, and III correspond to lineages *TAR*, *Angela* and *Ale*[27], because they contain *ATCOPIA95,BARE*-1, and *ATCOPIA4*, respectively.

The structural features of jatropha *copia*-type families were obviously related to lineages. The families of Lineage II were the longest, whereas those of Lineage III were the shortest. The families of Lineage III were also the shortest of LTR sequences (Figure 3 and Table 1). There were two additional nucleotides between LTR and the tRNA complementary sequence in PBSs of Lineages I and II, but not in those in Lineage III (Table 1). These results support our assertion based on phylogenetic data that these lineages are indeed derived from separate ancestral families.

### Copy number and presence of genes in flanking sequences of the *copia*-type elements

**Figure 5:**
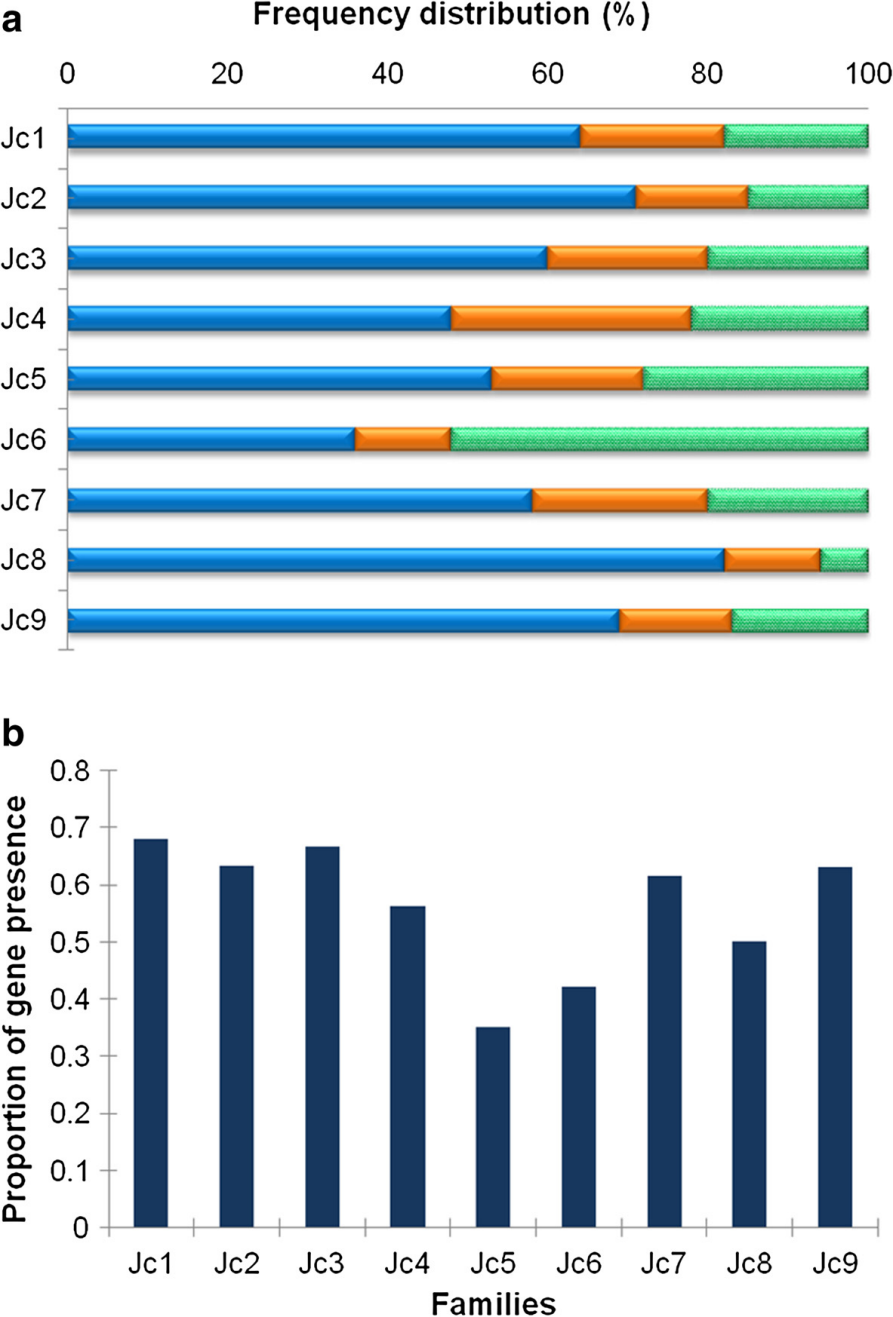
**Comparative analysis of copy number and presence of genes in flanking sequences among jatropha *copia*-type families. (a)** Distribution pattern of low (1–10 copies), moderate (11–100 copies), and high (over 100 copies) copy number flanking sequences in each family are represented in blue, orange and green, respectively. **(b)** Proportion of flanking sequences which contain gene(s) within 5 kb of the elements.

Full sequences of the *copia*-type families allowed us to define the flanking sequences of the elements. To develop a better understanding of the evolution and diversity of *copia*-type retrotransposons in jatropha, a search was applied throughout the jatropha genome database to examine the copy numbers of target sequences. As shown in Figure 5a, there seemed to be different preferences in copy numbers of target sequences among families. In *Jc1*, 2, 3, 8 and 9, more than 60% of the members existed in low-copy number regions, whereas more than 60% of *Jc6* members existed in high-copy number regions. Characterization of adjacent regions of retrotransposons provides an opportunity to find appropriate DNA markers and to understand the interaction of retrotransposons and genes. To address this issue, we further investigated the presence of genes in the flanking regions of the nine families by in-depth studying of the jatropha genome within 5 kb of the elements (Figure 5b). In *Jc1*, 2, 3, 7, and 9, more than 60% of flanking sequences had at least one gene. On the other hand, in *Jc5*, less than 40% of flanking sequences had gene(s). Thus, the families *Jc1*, 2, 3, 7 and 9 which existed in the gene-rich compartments of the jatropha genome were found to bear the highest potential as DNA markers. Moreover, analysis of the jatropha genome revealed that there were no obvious relationships between target copy numbers or presence of genes and lineages, unlike the structural features.

### Homogeneity of the *copia*-type retrotransposons distribution among Asian and African lines

**Figure 6:**
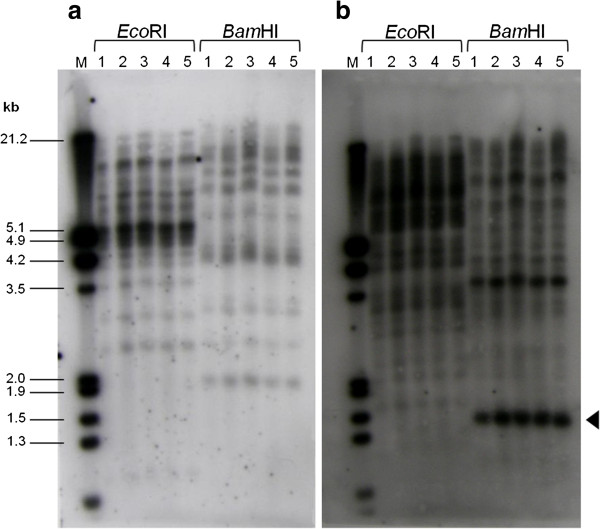
**Southern blotting analysis of the *copia*-type reverse transcriptase sequences.** Genomic DNA samples of Philippine, Chinese, Thai, Indonesian, and Ugandan lines (lanes 1 to 5) digested with *Eco*RI or *Bam*HI were hybridized with the RT probes of *Jc3 ***(a)** and *Jc5 ***(b)**. An arrowhead shows a *Bam*HI-digested fragment (ca. 1.5 kb) described in the text.

The distribution pattern of *copia*-type retrotransposons was compared by the genomic Southern hybridization among five jatropha lines (Philippine, Chinese, Thai, Indonesian, and Ugandan). Two RT sequences from families of different lineages (*Jc3* and *Jc5*) with moderate and high copy numbers (see Figure 2) were used as probes. As shown in Figure 6, the hybridization pattern using either of the two probes had no obvious variation among five lines. These results suggest that distribution patterns are almost the same among the five lines, which are consistent with previous results of SSR markers showing genetic homogeneity among Asian and African jatropha lines [11]. Hybridization using the *Jc5* probe showed an intense band of about 1.5 kb long in *Bam*HI-digested DNAs, which suggests that two *Bam*HI sites surrounding the RT region in the *Jc5* consensus sequence (see Figure 3) are conserved in a considerable number of the members.

### Insertion polymorphism among jatropha populations

**Figure 7:**
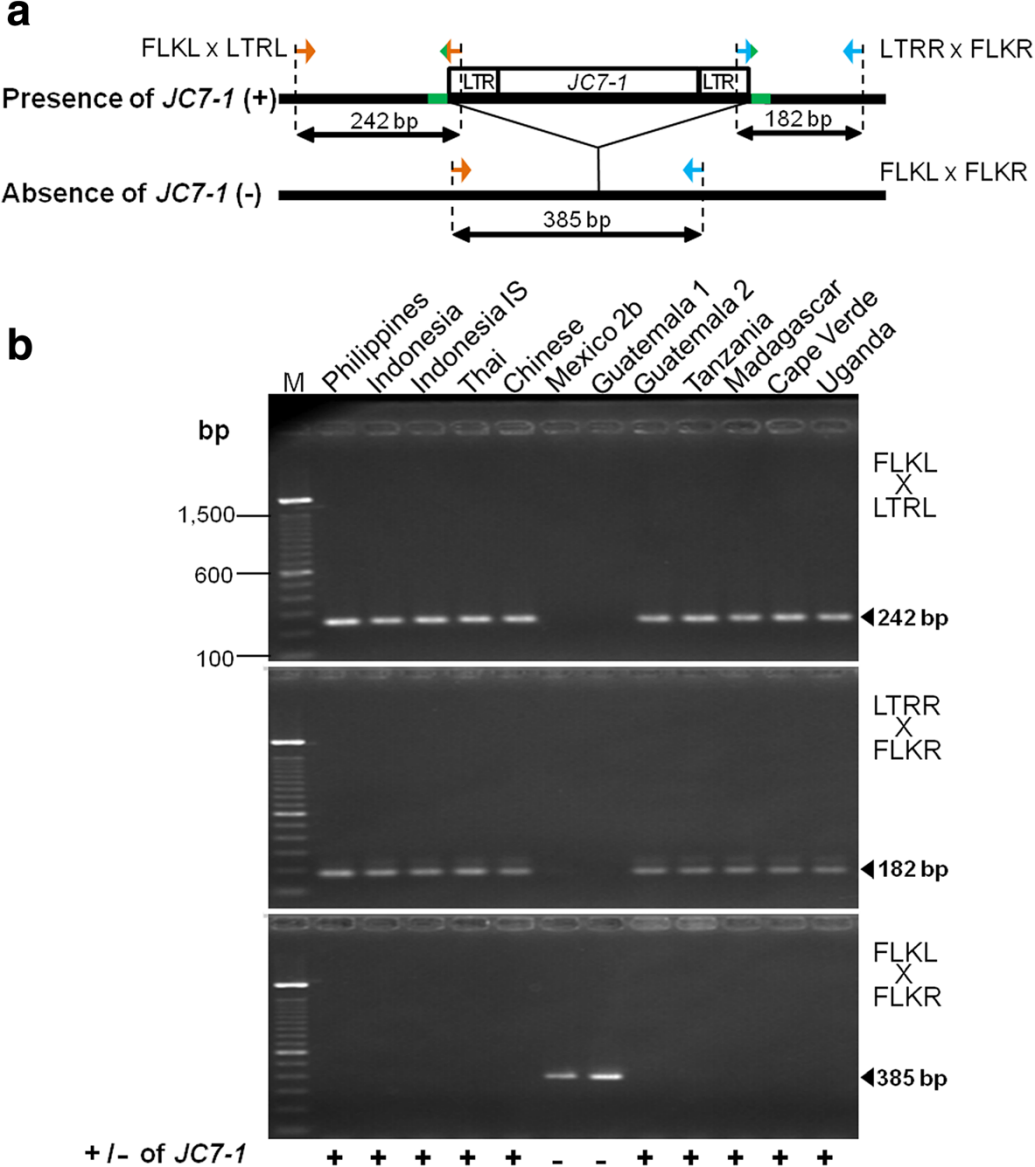
**PCR detection of the *JC7-1 *retrotransposon insertion polymorphism. (a)** Schematic illustration of RBIP primer design. Arrows indicate the position and direction of the primers used in this study, and target site insertion is represented in green. FLKL X LTRL: A primer set corresponding to the left flanking sequence (forward) and left LTR (reverse); LTRR X FLKR: A primer set corresponding to the right LTR (forward) and the right flanking sequence (reverse); FLKL X FLKR: A primer set corresponding to the left and the right flanking sequences. **(b)** Presence or absence of *JC7-1* restrotransposon marker in twelve jatropha accessions. PCR products are indicated by arrowheads.

An in-depth study of jatropha *copia*-type retrotransposons and their flanking DNA led to the acquisition of several recently-retrotransposed elements with identical LTR pairs, which are candidates of retrotransposon-based insertion polymorphism (RBIP) markers. Based on the sequence of LTRs and target site duplication (TSD), specific primer sets were designed to verify the presence or absence of *JC7-1*, one of the candidates of recently-retrotransposed elements in the *Jc7* family, among twelve jatropha populations from Asia, Africa, and the center of origin, Mesoamerica (Figure 7a). The PCR analysis of the *JC7-1 copia*-type retrotransposon showed that this marker has the ability to distinguish Mexico 2b and Guatemala 1 jatropha accessions from others (Figure 7b). *JC7-1* was not integrated to the genome of the aforementioned accessions at the individual insertion, whereas the other ten accessions including Guatemala 2 demonstrated its amplification (Figure 7b). This result indicates that the *JC7-1 copia*-type element was inserted into that locus, before the propagation of jatropha to the Africa and Asia from the center of origin, and Guatemala 2 is more closely related to Asian and African accessions than other two Mesoamerican accessions. The data presented here are consistent with the results of Southern hybridization (see Figure 6) and an identical distribution of the conserved RT region of *copia*-type retrotransposons among Asian and African jatropha (see Additional file 1). Moreover, it confirms previous reports about the close genetic relationship between Asian and African jatropha populations, which indicates that they share the same origin [13-15].

### Chromosomal locations of the jatropha *copia*-type retrotransposons

**Figure 8:**
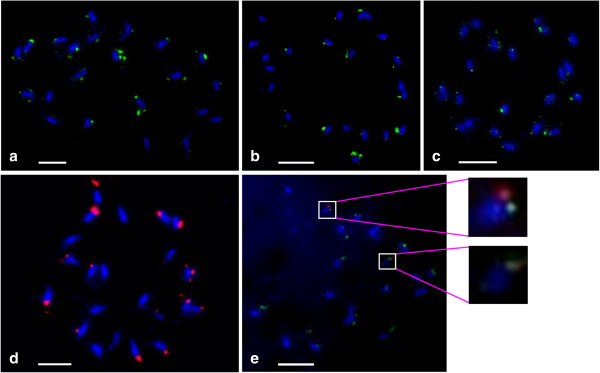
**Chromosomal distribution of jatropha *copia*-type retrotransposons.** Mitotic metaphase spread of *J. curcas* (2n = 22) after fluorescence *in situ* hybridization with RT element had high, moderate and low copy number as a probe. The chromosomes were counterstained light blue with DAPI. Green and red signal represent RT sequences labeled with DIG and biotin, respectively. **(a)** RT sequence belongs to family *Jc1* (high copy), **(b)***Jc5* (high copy), **(c)***Jc3* (moderate copy) and **(d)***Jc8* (low copy) were used as probes. **(e)** Double fluorescence *in situ* hybridization with the 5S rRNA gene sequence and the RT sequence of *Jc5*. Bars= 3 μm.

Determining the chromosomal locations of *copia*-type retrotransposons could contribute to the better understanding of the role and the dynamics of the repetitive elements in the genome and karyotype of jatropha, as well as facilitate the selection of families for informative markers. In order to gain insight into the chromosomal distribution of *copia*-type retrotransposons in the jatropha genome, FISH analysis was carried out using biotin- or digoxigenin (DIG)-labeled RT sequences as probes, which were selected from families of the three lineages that showed high (*Jc1* and *Jc5*), moderate (*Jc3*), and low (*Jc8*) copy numbers (Figures 8a-d). The *copia*-type retrotransposons of the all four families dispersed throughout the chromosomes but were predominantly located in the distal regions of chromosome arms. These results demonstrate the similar distribution patterns of *copia-*type retrotransposons in jatropha chromosomes among families of various lineages with different copy numbers. Interestingly, the intensities of FISH signals were obviously different among the chromosomes. This might be due to difference in copy numbers of the family members among the chromosomes. It was previously indicated that the genes for 5S rRNA were mapped at the terminal heterochromatin regions in two of the jatropha chromosomes [36]. Employing a double-label FISH assay to detect the physical distribution patterns of 5S rRNA genes and *Jc5* RT demonstrated that they are not overlapped, despite the presence in the distal part of chromosomes and intense hybridization signals of RT (Figure 8e). There is a good evidence for the similar distribution patterns of *copia-*type retrotransposons in jatropha chromosomes among families of various lineages with different copy numbers.

## Discussion

Genetic studies using molecular markers such as RAPD, AFLP, SSR, and SNP detected almost very low genetic divergence among *J. curcas* varieties cultivated currently in Africa and Asia [13-15]. In spite of a narrow genetic diversity among Asian and African accessions, significant climate- and practice-independent differences for various agronomic traits have been reported among and within these lines [37]. However, since the accessions showed mid-level epigenetic diversity, it is not still clear whether these variations result from genetic polymorphism. Hence, it is important to utilize other types of DNA markers to identify and characterize such accessions genetically, thereby enhancing the efficiency of breeding programs in jatropha populations. Retrotransposons are major components of the eukaryotic genome, which are supposed to be involved in diversity and evolution of species. Since several recent retrotransposition events are found in the plant genome [20], detecting the presence or absence of such retrotransposons is a suitable method to characterize population with low genetic diversity. Moreover, as revealed by pioneering studies on plant genomes, they afford several advantages (such as irreversibility and ubiquity) that make them very powerful tools as DNA markers for studying phylogenetic relationships and evolutionary history [38,39]. Development of retrotransposon-based insertion polymorphism (RBIP) markers requires characterization of retrotransposons. So far, however, retrotransposons have not yet been well characterized, nor have they been exploited as markers in jatropha.

In the present study, the degenerate primers corresponding to the conserved region of the reverse transcriptase were employed for genomic PCR to detect the *copia*-type retrotransposons in the genome of jatropha. In total, 157 sequences were isolated from Philippine, Chinese, Thai, Indonesian, and Ugandan lines. Consistent with high levels of heterogeneity reported among the RT sequences of *copia*-type retrotransposons in plants [40], most of isolated RT sequences differed from each other and distributed in 10 clusters, which corresponds to *copia*-type families of jatropha (see Figure 3). Distribution of isolated RT sequences among families showed similar distribution among the five lines (see Additional file 1). This is consistent with the similarity of DNA hybridization pattern of RT sequences among all lines (see Figure 6).

It has been reported that plant genome sizes are positively correlated with both the variation and copy number of LTR retrotransposon families, including *copia*-type retrotransposons [41]. Meanwhile, retroelements of Arabidopsis, which has a small genome size (130 Mb), accounts for less than 10% of the genome, whereas more than 75% of large-sized genome of maize (2.3 Gb) ascribed to the LTR retrotransposons [42]. In the case of the jatropha genome (370 Mb), we have previously reported that 36% of the genomic sequences were occupied by transposable elements in which *copia* and *gypsy*-type retrotransposons constituted major components by 8.0% and 19.6%, respectively [12]. The copy numbers of jatropha *copia*-type retrotransposon families identified in this study were different from each other. Therefore, presence of the families with high copy numbers (such as *Jc1*, 5, or 10) may have particularly affected the jatropha genome size.

Jatropha *copia*-type families identified in this study were classified into three lineages, which corresponded to three (*TAR*, *Angela*, and *Ale*) out of six lineages in *copia*-type families of other plant species. A data mining procedure from the jatropha genomic sequence using the BLAST algorithm from RT sequences of other *copia*-type families in other plant species, including those of the remaining three lineages (*Maximus*, *Ivana*, and *Bianca*), detected jatropha RT sequences that were not isolated using degenerate primers (data not shown). A lack of finding from these sequences using the PCR method suggests amplification biases of degenerate primers, which were also suggested by the distribution and the copy number analysis (see Figures 1 and 2). *Jc1*, one of the high-copy number families of jatropha, was closely related to *Tto1*, an active *copia*-type family of tobacco [33]. Because expression of *Tto1* is activated by various stresses, including viral infection or wounding [43], the question remains whether expression and retrotransposition of *Jc1* are activated by these stresses.

Preference of insertion sites was different among families in terms of the copy number of the insertion site and presence of flanking genes. For DNA markers, families that prefer to exist in gene-rich regions are more desirable. From this point of view, families *Jc1*, 2, 3, 7, and 9 are appealing candidates for the development of molecular marker systems in jatropha breeding. In the current study, polymorphism was detected by the presence or absence of the PCR products corresponding to the individual insertion site of the *JC7-1*, one of the selected marker candidates (see Figure 7). The presence of *JC7-1 copia*-type element in Guatemala 2, as well as in all Asian and African accessions, suggests that the retrotransposition of *JC7-1* is occurred in Mesoamerica before propagation to other continents. The data presented here revealed that this marker could detect genetic variation and the origin jatropha accessions that will be necessary for breeding programs. Interestingly, there was no identifiable relationship between the copy number of families and the presence of genes in the nearby flanking sequence (see Figures 2 and 5b), even though there is a possibility that the presence of retrotransposon might affect the expression of flanking genes epigenetically [44].

FISH on mitotic chromosomes showed jatropha *copia*-type families are dominantly clustered in the distal part of chromosomes (see Figure 8). Different localization of *copia*-type retrotransposons with 5S rRNA genes which present in the subtelomeric hetrochromatin regions indicates that they accumulate in the gene-rich euchromatic regions of jatropha chromosomes. This kind of distribution pattern has been shown to be typical for *copia*-type retrotransposons, such as *Matita* in peanut [21]. Moreover, these observations were substantiated by database analysis of the flanking regions (see Figure 5).

The chromosome-specific hybridization pattern will facilitate the identification of individual chromosomes, a suitable cytogenetic approach considering that jatropha chromosomes are mostly metacentric and of similar size. The aforementioned features of chromosomal localization were similar among four families examined, regardless of lineage or copy numbers. This suggests that preferences of localization in the chromosome scale are almost same among families, although those in a small scale (within 5 kb) are different from each other.

Retrotransposon-based insertion polymorphism (RBIP) markers can be distributed extensively enough to support genetic mapping and genetic diversity studies among and within organisms, even for populations suffering from narrow genetic base [45]. We established an RBIP system for profiling jatropha accessions using *JC7-1* as a DNA marker. This is a simple technique that can easily be executed by PCR following gel electrophoresis, or even by means of automated high-throughput gel-free procedures such as TaqMan or DNA chip technology [22]. This system shows high potential to address the evolution issues in the jatropha genome in its places of origin. Moreover, LTR and flanking sequences obtained here can be used to develop other retrotransposon-based molecular markers, including the sequence-specific amplification polymorphism (SSAP) marker system and inter-retrotransposon amplified polymorphism (IRAP) [46]. The information obtained herein will be applicable to developing further RBIP marker sets in jatropha to enucleate evolutionary and the genetic relationships among its various accessions.

## Conclusions

This is the first extensive study to perform a genome-wide survey of the structure, phylogenetic diversity and chromosomal distribution of *copia*-type retrotransposons in the biodiesel crop, jatropha. Combining a molecular genetic approaches and a computer-based data mining, we have isolated and characterized ten *copia*-type retrotransposon families, which were then grouped into three lineages. The representation and dynamics of the ten *copia*-type families were further revealed by comparative analysis of copy number and presence of genes in their flanking sequences in the jatropha genome, and finally *copia*-type families *Jc1*, 2, 3, 7, and 9 were found as noteworthy candidates for the development of DNA marker systems in jatropha. We introduced *JC7-1* as a specific RBIP marker that is which appears suitable enough to differentiate certain Mexican and Guatemalan accessions from others. Given the presence of *copia*-type retrotransposons in the gene-rich regions of the jatropha genome based on database analysis of the flanking sequences, the FISH patterns also confirm that the retrotranspsons of this kind were dispersed throughout all chromosomes with clustering dominantly in the distal part of chromosome arms. Altogether, the findings of the present study indicate that *copia*-type retrotransposons can be exploited as a powerful molecular marker system in jatropha breeding programs.

## Methods

### Plant materials

Seeds of five *Jatropha curcas* L. accessions from distinct geographic areas of the Philippines, China, Thailand, Indonesia and Uganda were collected and planted at the environmentally controlled glasshouse of Osaka University (Suita, Japan). For fluorescence *in situ* hybridization (FISH) experiment, jatropha seeds of the Philippine line were peeled out and germinated in moist tissue paper at 30°C.

### Isolation of genomic DNA, PCR amplification and cloning of RT sequences

Total genomic DNA was extracted from young leaves based on CTAB protocol [47] in 2% Cetyltrimethylammonium bromide (CTAB), 100 mM Tris–HCl (pH 9.5), 20 mM EDTA, 1.4 M NaCl and 1% β-mercaptoethanol. Three degenerate primer sets corresponding to highly conserved peptide sequence of *copia*-type reverse transcriptase (RT), F: “5′- ACNGCNTTYYTNCAYGG-3′ and 5′- ARCATRTCRTCNACRTA-3′” [30], V: “5′- CARATGGAYGTNAARAC-3′ and 5′- CATRTCRTCNACRTA-3′” [32], and H: “5′-GAYGTNAARACNGNTTYYT-3′ and 5′- AYRTRTCNACRTANARNA-3′” [31] were used. The PCR reaction was carried out in a 50 μl reaction mixture containing 100 ng genomic DNA, 250 μM dNTPs, 5 μl 10× ExTaq buffer, 20 pmol of each forward and reverse, along with 2.5 units of Ex Taq polymerase (Takara, Japan) on TaKaRa PCR thermal cycler Dice (Takara, Japan). PCR conditions included an initial denaturation step of 96°C for 5 min followed by 30 cycles of 96°C for 30 s, 50°C, 45°C or 47°C for 1, 1.5 or 1 min for F, V and H primer set, respectively, followed by a final elongation step at 72°C for 5 min. The desirable bands were purified with the Wizard SV gel and PCR clean up system (Promega, USA) and, 2.5 ng of purified PCR product was treated by 400 μM dNTPs and 0.15 units Ex *Taq* polymerase (Takara) in 5 μl total volume at 55°C for 30 min. The fragments were then cloned into the pGEM-T Easy vector (Promega) using T4 DNA ligase (Takara), according to the manufacturer’s instructions and then, transformed into the *E. coli* DH5α followed by screening white colonies in selective LB/IPTG/X-gal/Ampicillin/agar plates. Identity of positive recombinant clones was further verified by colony-PCR. DNA sequencing was performed on an ABI PRISM 3100 DNA Sequencer (Applied Biosystems, US) using SP6 primer and BigDye terminator ver. 3.1.

### Computer analysis of sequences

The initial nucleotide sequences of PCR amplified fragments were trimmed and amino acid sequences were deduced using GENETYX-MAC ver. 13 (GENETYX Corporation, Japan) with a consideration of spontaneous frameshift mutations and subsequently multiple sequence alignment was conducted by ClustalW ver. 1.83 online software (http://clustalw.ddbj.nig.ac.jp/top-e.html). Phylogenetic analyses of the aligned RT sequences were conducted using MEGA ver. 4 software [48]. Difference in family distribution of clones was evaluated by Chi-square test. The RT consensus sequence of each family was deduced based on multiple alignments of cloned RT sequences. Relative copy number of each family was estimated by the BLAST algorithm using consensus RT sequences as queries against the Jatropha Genome Database available at the Kazusa DNA Research Institute (http://www.kazusa.or.jp/jatropha/). Sequence similarity with an e-value of less than e^-20^ was considered significant, and hit numbers were counted.

Full consensus sequences of the families by BLAST search of the Jatropha Genome Database using consensus RT sequences as queries. Contigs with high BLAST were aligned using GENETYX-MAC and Harrplot ver. 2 (GENETYX Corporation, Japan), and consensus sequences of them were deduced. BLAST search and alignment were then performed again using the new consensus sequences as queries until they reached both ends of the elements. The nucleotide sequences of the *copia*-type retrotransposon families were used to obtain GAG-POL amino acid sequences. The most conserved RT regions of GAG-POL (ca. 220 amino acids) were aligned with corresponding sequences of *copia*-type retrotransposons in other species, and a neighbor-joining tree was constructed using ClustalW software.

To estimate copy numbers of the target sequence of jatropha *copia*-type elements, 500 bp sequences flanking to the elements were used as queries in the BLAST search, and the hit numbers (< e^-20^) were counted. To acquire the frequency of the gene existence nearby retrotransposons, we searched presence of genes in flanking sequences within 5 kb of the elements. Twenty or more flanking sequences were analyzed per family for both analyses.

### RBIP primer design and PCR analysis

Three primer sets were designed to amplify specific junction regions between LTR and flanking sequences of *JC7-1* at the particular insertion site (Table 2 and Figure 7a). Genomic DNA samples of twelve jatropha accessions taken from Africa, Asia and Mesoamerica were investigated. If *JC7-1* is present at the insertion site, the first primer set amplifies a 242 bp fragment of the upstream flanking sequence of *JC7-1*, and the second primer set amplifies 182 bp downstream fragment of the flanking sequence of this retrotransposon marker. The third primer set was specific to the 5′ and 3′ flanking regions to score the corresponding empty site. PCR amplifications were performed in a 5-μl reaction mixture containing 0.5 ng of genomic DNA, 20 pmol each of forward and reverse primers, 200 μM of each dNTPs, 0.3 μl of 50 mM MgCl_2_, 0.5 μl of 10× NH_4_ buffer, and 0.04 U of BIOTAQ DNA polymerase (Bioline, UK). Reactions were denatured at 94°C for 2 min, followed by 35 cycles of 45 s at 94°C, 45 s at 55°C and 2 min at 72°C, with a final elongation step of 10 minutes at 72°C. The amplified PCR products were separated by electrophoresis on 2% Agarose gels in a 1× TAE buffer and visualized under UV light after staining with ethidium bromide.

**Table 2:** **List of the primers for *JC7-1 *RBIP detection**

**Name**	**Primer sequence (5′-3′)**
FLKL	CAAAGCACACGAGGATTCAG
FLKR	CAGGTCCAAATCTCCTCGTG
LTRL	GAAAATTAAATCCAACATGT
LTRR	GAGATTAATTCTTAACAGAA

### Southern hybridization

About 10 μg of genomic DNA samples of five accessions were digested with *Bam*HI and *Eco*RI individually and fractionated on 1% agarose gel. These gels were Southern blotted on Amersham Hybond-N+ membrane (GE Healthcare, Amersham, UK) as described by Sambrook et al. [49]. Two DIG-labeled DNA probes were constructed from the isolated RT domain of *Jc3* and *Jc5* using a PCR DIG Probe Synthesis Kit (Roche Diagnostics, Mannheim, Germany). The membranes were pre-hybridized using DIG Easy Hyb (Roche Diagnostics, Mannheim, Germany), followed by hybridization with DIG-labeled probes according to the protocol of the DIG High Prime DNA Labeling and Detection Starter Kit II (Roche Diagnostics, Mannheim, Germany). The hybridized probe was visualized on an X-ray film using CSPD chemiluminescence substrate (Roche Diagnostics, Germany).

### Fluorescence *in situ* hybridization (FISH)

Meristem of root tips of jatropha seedlings were soaked in 2 mM 8-hydroxyquinoline solution for 3 h to accumulate metaphase and then fixed in 3: 1 (v/v) ethanol: acetic acid. The fixed root were washed in water and digested in enzyme solution (2% Cellulase and 5% Pectolyase) at 37°C for 45 min. Cells were squashed on the slide in 15 μl of 3:1 (v/v) ethanol: acetic acid. A PCR product set of the isolated RT domain of *J. curcas* were labeled with biotin-16-dUTP using a Nick-translation kit with biotin-16-dUTP (Roche Diagnostics, Mannheim, Germany) and digoxigenin (Roche Diagnostics, Mannheim, Germany), and used as probes for in situ hybridization experiments. FISH was done as described by Ohmido and Fukui [50] with minor modifications. The 5S rRNA probe was amplified and labeled with biotin-16-dUTP (Roche Diagnostics, Mannheim, Germany) by the PCR labeling method for simultaneous hybridization [36]. The denaturing solution consisted of 70% formamide in 2× SSC and 1 μg/ml labeled probe. The mixture was added onto slides and denatured at 70°C for 4 min. Dehydration was performed in cold ethanol and slides were then air dried at room temperature. The probe mixture including hybridization buffer (50% Formamide, 2× SSC, 10% Dextran sulfate) and 100–200 ng labeled probe after denaturation at 85°C for 10 min was applied to each slide and incubated at 37°C overnight in a humid dark box. The sites of probe hybridization were detected by streptavidin-Cy3 (Jackson ImmunoResearch Laboratories, West Grove, PA) and anti-digoxigenin-FITC (Roche Applied Science), and chromosomes were counter-stained with 1 μg/mL 4, 6 diamidino-2-phenylindole (DAPI) in VectorShield (Sigma-Aldrich, St. Louis, MO). The slides were observed under an Olympus BX50 fluorescence microscope, and the images were captured with a CCD camera (Olympus DP70) followed by processing by Image J software.

## Abbreviations

DIG: Digoxygenin; FISH: Fluorescence *in situ* hybridization; LTR: Long terminal repeat; ORF: Open reading frame; PBS: Primer binding site; PPT: Polypurine tract; RBIP: Retrotransposon-based insertion polymorphism; RT: Reverse transcriptase.

## Competing interests

The authors declare that they have no competing interests.

## Authors’ contributions

AA participated in the design of the study, performed the experiment and drafted the manuscript. ST contributed in the experimental design, a part of computer analysis, checking the results and edited the manuscript. HS commented on the manuscript and NO provided technical advices for FISH experiments. KF participated in the design of the research plan, contributed to the manuscript edition, and obtained funding for the research work. All authors read and approved the final manuscript.

## Supplementary Material

Additional file 1 Distribution of isolated RT sequence. Click here for file
