# Supplementary figures and images for: Structural characterization of copia-type retrotransposons leads to insights into the marker development in a biofuel crop, Jatropha curcas L
Source: Biotechnol Biofuels. 2013 Sep 10;6:129. doi: 10.1186/1754-6834-6-129 (PMC3852365; doi:10.1186/1754-6834-6-129)

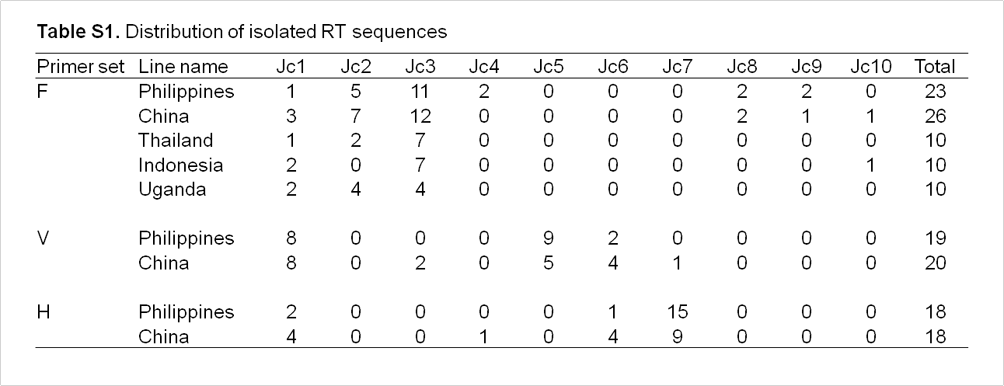

Supplement: Additional file 1 — Distribution of isolated RT sequence. [file 1754-6834-6-129-S1.tiff]
